# Barriers and facilitators of integration of pharmacists in the provision of clinical pharmacy services in Tanzania

**DOI:** 10.1186/s12875-023-02026-4

**Published:** 2023-03-17

**Authors:** Manase Kilonzi, Ritah F. Mutagonda, Hamu J. Mlyuka, Dorkasi L. Mwakawanga, Wigilya P. Mikomangwa, Wema A. Kibanga, Alphonce Ignace Marealle, Bertha Mallya, Deogratias Katabalo, Sofia Sanga, Fredrick Kalokola, John Rwegasha, Rose Magambo, John Mmassy, Sungwa Kabissi, Josephine A. Balati, Peter Maduki, OmaryMashikuMinzi, Appolinary A. R. Kamuhabwa

**Affiliations:** 1 School of Pharmacy, the Muhimbili University of Health and Allied Sciences, P. O. BOX 65013, Dar Es Salaam, Tanzania; 2 School of Nursing, the Muhimbili University of Health and Allied Sciences, P. O. BOX 65001, Dar Es Salaam, Tanzania; 3 School of Pharmacy, the Catholic University of Health and Allied Sciences, P. O. BOX 1464, Mwanza, Tanzania; 4 Department of Internal Medicine, Muhimbili National Hospital, P. O. BOX 65000, Dar Es Salaam, Tanzania; 5 School of Medicine, the Catholic University of Health and Allied Sciences, P. O. BOX 1464, Mwanza, Tanzania; 6 Christian Social Services Commission (CSSC), P.O BOX 9433, Dar Es Salaam, Tanzania

**Keywords:** Barriers, Facilitators, Clinical pharmacy services, Integration, Pharmacists, Tanzania

## Abstract

**Background:**

Collaboration between medical doctors and nurses in the provision of healthcare services has been there for decades. The concept of clinical pharmacy services as a main goal for pharmacy practice is relatively new and is yielding more positive results for healthcare providers (HCPs), patients, and the health system. This study assessed barriers and facilitators toward the integration of pharmacists in the provision of CPS in Tanzania.

**Methods:**

A qualitative study was conducted in five tertiary hospitals representing Tanzania mainland. Ten (10) focus group discussions (FGDs) with 83 HCPs and 14 in-depth interviews (IDIs) with hospital administrators in referral hospitals were conducted between August and September 2021. The experienced qualitative researchers moderated the IDIs and FGDs, and all discussions were audio-recorded. Finally, the audios were transcribed verbatim, and analysis was done using a thematic approach.

**Results:**

Limited skills, lack of confidence, poor communication, inferiority, and superiority behaviors among HCPs were among the mentioned barriers. Shortage of pharmacists, lack of in-job training, standard operating procedures (SOPs), and guidelines were also mentioned. The study noted the high acceptability of CPS by other HCPs, the positive perception of pharmacists, and the recognition of CPS by the Tanzania Pharmacy Act and regulation.

**Conclusion:**

The facilitators and barriers to the integration of pharmacists in the provision of CPS lie at the individual, health facility, and health system levels. Therefore, the study recommends in-job pharmacists training, fostering teamwork among HCPs, and development of CPS SoPs, and guidelines.

## Background

The provision of quality healthcare services requires the participation of all healthcare professionals in teamwork to benefit the patient [1]. Traditionally, medical doctors and nurses have directly provided care and treatment services to patients. Unfortunately, the traditional approach no longer fits the six healthcare quality improvement goals developed by the Institute of Medicine (IOM) [2]. The IOM advocates a team-based approach which is associated with several benefits to patients, HCPs, and the health system. The reported benefits are shortened average hospital stay, reduced chances of readmission, increased community trust in the healthcare system, improved service quality, and reduced morbidity and mortality [3, 4]. Pharmacist integration into the team is a relatively new concept, however when done it seems to benefit patient care [4].

Integrating pharmacists into the provision of healthcare services in developed countries commenced some years ago when the focus of the pharmacy profession shifted from drug product to the patient [5–7]. Among the benefits of involving pharmacists include detecting drug therapy problems and developing various chronic disease indicators (CDIs) (example of the CDI are glycosylated hemoglobin and dilated eye examination for diabetes) [8, 9]. However, even in these developed countries where CPS is well established, studies have outlined several barriers and facilitators to providing the services [1, 10]. Lack of pharmacist role definition, absence of an established relationship of trust and respect with existing team members, inadequate pharmacist training, a need for mentorship or peer support, pharmacist personality, resources and funding, and a lack of adequate space were reported to affect provision of CPS [1, 8, 11].

In Africa and Asia, the barriers to the provision of CPS may be grouped as external, financial, and individual-related factors [12]. External factors include lack of policies, poor leadership support, and shortage of staff. Financial factors are associated with a lack of monetary incentives and in-job training, while individual-related factors include inadequate clinical and communication skills, medical doctors’ dominance and opposition, lack of confidence, assertiveness, negative attitude, and mindset among pharmacists towards CPS provision [12–14].

To ensure multidisciplinary provision of healthcare in Tanzania. In 2018, Ministry of Health, through the Pharmacy Council, has revised pharmacists’ professional regulations requiring all pharmacists to participate in the provision of CPS. To be in line with the government directives, the medical universities in Tanzania amended their curriculum to include the participation of bachelor of pharmacy students in the provision of CPS with other medical professions, especially during major and service ward rounds. In addition, the Muhimbili University of Health and Allied Sciences (MUHAS) has established a Master of Pharmacy degree in Hospital and Clinical pharmacy to equip pharmacists with more knowledge and skills in CPS provision.

Despite the efforts mentioned above to ensure the integration of pharmacists into the provision of CPS in Tanzania, the participation of pharmacists is still suboptimal. Therefore, this study was conducted to explore the barriers and facilitators towards the integration of pharmacists into the provision of CPS in Tanzania.

## Methods

### Study designs

This was a qualitative study designed to explore the barriers and facilitators to the provision of CPS in Tanzania. The study was conducted between August and September 2021. In-depth interviews (IDIs) and focus group discussions (FGDs) with hospital administrations and HCPs were conducted to explore their perspectives on CPS provision in Tanzania.

### Study settings

The study was conducted in 4 zonal referrals and 1 national hospital, which are Bugando Medical Center (BMC), Mbeya Zonal Referral Hospital (MZRH), Kilimanjaro Christian Medical Center (KCMC), Benjamini Mkapa Hospital (BMH), and Muhimbili National Hospital (MNH). The hospitals were selected because they serve as tertiary hospitals in Tanzania and have a relatively adequate number of staff, including pharmacists. Besides, these hospitals serve the majority of Tanzanians and nearby countries; in average each hospital serve around 1,000 outpatients and 700 inpatients.

### Sampling technique, sample size, and study population

A purposeful sampling technique was used to select the participants based on their administrative position, level of education, clinical involvement in the direct provision of care and treatment to patients, and years of experience. In each hospital, the Executive Director, Director of Medical Services, and the Heads of the Department of Pharmacy were subjected to IDIs totaling 15 IDIs in 5 hospitals. We also conducted two FGDs, one with pharmacists only and another with nurses and medical doctors per hospital making 10 FGDs for all sites.

### Data collection procedure

The semi-structured interview guides for IDIs and FGDs were developed in English and then translated into Swahili. These interview guides were developed based on the objectives, a good literature review, and the researchers’ experience. The guides were improved based on emerging issues as we moved on with interviews. The guides were composed of open-ended questions and probes that explored information on the barriers and facilitators toward the provision of CPS in the respective hospitals. The probes explored individual-related, health facility-related, and health system-related barriers and facilitators toward the integration of pharmacists in the provision of primary healthcare services.

All IDIs and FGDs were conducted by four researchers (two per interview) with experience in qualitative data collection. Among the researchers involved in data collection; two have master of philosophy in pharmacology and therapeutics, one have master of pharmacy in hospital and clinical pharmacy and the last have master of nursing in midwives. During the interview, one interviewer moderated the discussion, and another one was responsible for taking notes on key issues, including non-verbal communication. Before the beginning of the interview, the important demographic information of the study participants was collected (age, gender, cadre, highest professional education level, and working experience). Hospital administrators were visited in their offices within the hospital for the interviews based on the prior set of appointment. FGDs with pharmacists, nurses, and medical doctors were conducted in a quiet designated room at the respective health facility at their convenient time. All interviews and discussions were audio-recorded. Before each IDI and FGD, written informed consent was provided by participants after explaining the purpose of the study and that the session would be audio-recorded. Each FGD consisted of 6 to 12 participants. FGDs and IDIs lasted between 60 to 120 and 30 to 60 min, respectively. Moreover, similar information started to reappear after visiting 4 hospitals, however, data saturation was not formally addressed.

### Data analysis

The audio-recorded interviews and discussions were first transcribed verbatim in the Swahili language. Six researchers were given transcripts and field notes to read and re-read to be familiar with the data and context and get a general understanding of the participants’ accounts before the analysis. The data were analyzed using a hybrid thematic analysis approach using inductive and deductive reasoning. The research team was composed of members with various health and social sciences backgrounds. These were midwifery specialists, qualitative researchers, sociologists, and public relations experts, while the rest were pharmacists with specializations in clinical pharmacy, pharmacology, and therapeutics. Then, the codebook with initial codes was developed deductively from existing domains and inductively from emergent ideas noted during familiarization with data. The open coding was done in pairs to ensure inter-code reliability allowing the discussion of discrepancies and disagreements that were then resolved. Agreements were reached with the team. The codebook was revised as we moved on with the analysis process. The identified codes list was then observed for commonalities and differences and put into sub-themes. Subthemes were collated, and repeated patterns identified across the data set were identified as themes. Themes were refined and finalized by reviewing and discussing them with the entire team of researchers. Lastly, the generated sub-themes and themes were presented with quotes describing each theme's meaning.

## Results

A total of 97 participants were interviewed. Out of 97, 14 were hospital administrators (Executive Directors (4), Directors of Medical Services (5), and Heads of Department of Pharmacy (5)), and the remaining 83 were HCPs comprised of pharmacists, medical doctors, and nurses. Following data analysis, we managed to explore barriers and facilitators towards the provision of CPS in Tanzania, as shown in Table 1 below.

**Table 1:** Barriers and facilitators towards the provision of CPS in Tanzania

Barriers to CPS provision in Tanzania	Facilitators of CPS provision in Tanzania
1.Individual-related barriers ✓ Limited skills and lack of confidence among pharmacists in CPS provision ✓ Poor communication among HCPs ✓ Superiority and inferiority behavior among HCPs 2.Health facility-related barriers ✓ Insufficient number of pharmacists in hospitals ✓ Lack of in-job building capacity platforms for pharmacists on CPS ✓ Less prioritized services in most healthcare facilities 3.Health system-related barriers ✓ Lack of guidelines and SOPs on CPS provision ✓ Few pharmacists are allocated per facility in the scheme of services	1.Individual efforts to implement CPS ✓ Acceptability of CPS by other HCPs ✓ Positive perception of pharmacists towards the provision of CPS ✓ Competency of pharmacists on CPS provision 2.Health system efforts to implement CPS ✓ Reliable hospital administration support ✓ Recognition of CPS by the Pharmacy Act and Regulation in Tanzania

### *Barriers* to CPS provision in Tanzania

1. **Individual-related barriers**
  - ***Limited skills and lack of confidence among pharmacists in cps provision***
    Pharmacists, nurses, and medical doctors who participated in the idis and fgds responded that pharmacists have limited knowledge and skills about the provision of cps. some participants responded that pharmacists have limited skills in patient clerkship, taking vital signs, and dose preparation affecting their involvement in the primary care team.
    *“The challenge that we have is that pharmacists are not used to this kind of service, so it’s hard for them to participate and sometimes they are scared of being challenged when asked questions. so if they face challenges, they lose hope don’t agree to go back again.” national hospital head of pharmacy unit.*
    *Participants said pharmacists lack the confidence to speak in the middle of other hcps and others are afraid of facing challenges of questions and comments from the team, which is composed of different hcps. participants also responded that pharmacists are reluctant to update their knowledge on how various diseases are currently managed, affecting their participation in the primary care team.*
    *“another big problem is that health care professionals in other areas are not confident, and that lack of confidence is self-made. i have observed that people will listen to you, but also look at your confidence level and whether you are sure of what you are telling them. so, there is a fear of being ashamed.” national hospital director.*
  - ***Poor communication among hcps***
    Participants said that the lack of positive relationships and collaboration among hcps affects the involvement of pharmacists in the provision of cps. one of the medical doctors said that the perception that wards in the hospitals are for doctors and nurses limits the involvement of other healthcare professionals including pharmacists, in the participation of major and service ward rounds. participants further insisted that some medical doctors and nurses doubt pharmacists' competency in providing cps. some pharmacists during fgds pointed out that some medical doctors are arrogant and use bad language when responding to issues raised by other hcps in the primary care team.
    *“There’s one who went to the ward round, and one among the people who were in the round said, “this little pharmacist is the one we have for today?”. the pharmacist felt very bad and couldn’t go on with the ward round. so, i think when you will be setting these policies, they first must understand that pharmacists do not only dispense drugs.” national hospital pharmacist.*
  - ***Superiority and inferiority behavior among hcps***
    participants said medical doctors are so arrogant when it comes to patient management, and they think they know more than anyone else. due to this attitude, some junior medical doctors, pharmacists, and other healthcare professionals fear embarrassment when participating in primary patient care services. participants added that sometimes a medical doctor might pose a difficult question in front of the patient and the team to embarrass the pharmacist. moreover, participants said because pharmacists are not used to cps provision some demonstrate inferiority, and if they fail to respond to the medical doctor’s questions, they become discouraged from participating in the provision of cps.
    *“First, there is a challenge between health care providers themselves; doctors as they interact with other cadres. personality issues hinder us the most, as one perceives that what they say is the only thing that must be followed, nothing else. also, there is a tendency to see that other cadres know nothing; we are the ones who know more.” central zone hospital medical doctor.*
2. **Health facility-related barriers**
  - ***Insufficient number of pharmacists in hospitals***
    pharmacists, nurses, and medical doctors said there are few pharmacists in their hospitals. participants said that due to the shortage of pharmacists in the hospitals, they are overwhelmed by other responsibilities. due to the shortage of hcps, sometimes pharmacists are assigned to oversee non-pharmaceutical tasks by the administration, which further limits their involvement in the provision of cps in the hospitals.
    *“The main reason for the lack of participation of pharmacists in the provision of cps is that, despite being ready and committed, there is a shortage of staff, so you may find that you have a lot of duties that you must perform as a pharmacist. therefore, shortage of staff, especially pharmacists is a major problem for effective provision of cps in the hospitals.” northern zone hospital head of pharmacy unit.*
  - ***Lack of in-job building capacity platform for pharmacists***
    pharmacists mentioned that there is no comprehensive plan for on-job training to build their capacity to provide cps in the hospitals. some pharmacists claimed that they are yet to attend any training since they started working in their current hospitals. pharmacists insisted that lack of on-job training affects their involvement in the provision of cps.
    *"Those in the hospitals should be trained to get new exposure. usually, pharmacists are forgotten in fellowship programs to update their knowledge, including the provision of cps. most of the fellowship programs include only doctors and nurses." central zone hospital head of pharmacy unit.*
  - ***less prioritized services in most healthcare facilities***
    Participants said cps is prioritized less in most of the healthcare facilities. the participants noted that there is no job description regarding cps. there are no standard operating procedures (sops) and clear schedules to guide the provision of cps during ward rounds. there is also no dedicated space in the patients` files for pharmacists to document their suggestions about the prescribed medicines. pharmacists said that they have a poor working environment and that the available pharmacies in the hospitals have inadequate physical space for pharmacists to provide the appropriate services, including patient counselling on pharmacotherapy. in addition, limited mentorship and supportive supervision for pharmacists affect the involvement of pharmacists in the provision of cps.
    *“They have given pharmacists the responsibility of ensuring that medicines are properly utilized to generate financial resources for the hospital instead of basing on their primary responsibilities such as participation in ward rounds to propose medications and discuss treatment plans with the doctors and nurses. the current practice is that a pharmacist will only verify if the health insurance forms are filled based on the procedure, what medications were used, and what investigation was done. they are the ones who will sum up and submit to the organization concerned the insurance receipts for payments.” central zone hospital medical doctor.*
3. **Health system-related barriers**
  - ***Lack of guidelines, sop, and monetary motivation on cps provision***
    Participants said that the lack of enforcement from the ministry of health and the respective authorities for pharmacists to participate in the provision of cps limits the services. participants said that the lack of sops and guidelines for the provision of cps services affect pharmacists’ involvement in primary healthcare services. pharmacists with masters in hospital and clinical pharmacy mentioned that they are not recognized in the scheme of services for healthcare workers as specialists by the ministry of health. as a result, they are not remunerated according to their qualifications compared to medical doctors and nurses. this is one of the reasons why many pharmacists are unwilling to undergo postgraduate training, including hospital and clinical pharmacy that would have supported the effective implementation of cps in the hospitals.
    *"But also another thing is recognition in the scheme, i have studied clinical pharmacy, but i am paid the same salary as a pharmacist with a bachelor’s degree. so, it will require a clinical pharmacist to have passion for the provision of cps, but there is no added remuneration in terms of salary. as human beings, most of us advance in our academic careers because, among other motivating factors, we want to benefit in terms of salary, which is not the case for pharmacists with postgraduate degrees in the health sector in tanzania.” central zone hospital head of pharmacy unit.*
  - ***Few pharmacists are allocated per facility in the scheme of services***
    The scheme of services for pharmacists in tanzania indicates that a regional referral hospital can fully operate with a maximum of 4 pharmacists. participants said that this number is too small for them to perform all the pharmaceutical activities, including the provision of cps in the hospitals. the lack of harmonized guidelines for the provision of cps affects the participation of pharmacists in primary healthcare services. moreover, the lack of dedicated space for documentation of pharmacists’ interventions in the patient file limits pharmacists’ involvement in providing primary healthcare services.
    *“There are a lot of graduates with a pharmacy degree, but only a few are employed, so they cannot apply what they have studied to improve the quality of services in hospitals. the employers have not yet solved what is needed because most of the pharmaceutical employees are diploma and certificate holders because they think that the main responsibility of pharmacists in the hospitals is only dispensing drugs. this is a misconception because pharmacists are not only supposed to dispense” central zone hospital medical doctor.*

### Facilitators of CPS provision in Tanzania

1. **Individual efforts to implement CPS**
  - **Acceptability of CPS by other HCPs**
    Nurses and medical doctors agreed that CPS is a good service, and they are ready to work as a team with pharmacists. Participants demonstrated a good attitude towards CPS. Nurses and medical doctors said they need CPS while attending patients during major and services ward-rounds and in clinics. Furthermore, nurses and medical doctors indicated they were ready to accommodate CPS in their working areas.
    *“It is something we have been lacking for a long time because pharmacists are an essential part of the provision of patient care and their presence is required.” National Hospital Clinical Nurse.*
  - **Positive perception of pharmacists towards the provision of CPS**
    Pharmacists mentioned that CPS is among their core duties, and they are willing to participate in the provision of primary healthcare services directly to patients.
    *“Saying that high workload is the primary reason for us not participating in the ward rounds is not a good reason. Provision of CPS is our primary obligation and we must fulfill this.” Southern Zone Hospital Pharmacist.*
  - **Competency of pharmacists for provision of CPS**
    Pharmacists who participated in this study said they have adequate clinical exposure, knowledge of pharmacotherapy, and good communication skills to offer CPS.
    *“As I have said, pharmacists come to the wards and they are very competent. The main challenge they face is the high workload.” National Hospital Medical Doctor.*
    *“I see them with good communication skills, able to communicate with the prescribers regarding the prescribed medications. Their good rapport helps them to interact well with prescribers.” Central Zone Hospital Pharmacist.*

2. **Health system efforts to implement CPS**
  - **Reliable hospital administration support**
    Pharmacists who participated in this study said they received adequate support from the hospital administration. Some participants said that the Head of the Pharmacy Department always encourages them to attend major and service ward rounds with other HCPs. Participants said that the hospital administration has allowed them to access patient files and other related documents to add their contributions to the prescribed medications. In some settings, participants said the administration encourages pharmacists to attend clinical meetings for learning and familiarization with patients before participating in the ward rounds. In some hospitals, the provision of CPS has been added as one of the hospital performance indicators. Some participants added that the administration hires pharmacists through short-term contracts to expand pharmaceutical care services in their facilities. This has enabled to increase the number of pharmacists as well as improved the provision of CPS.
    *“We have tried to encourage each other and added it in the pharmacists’ open performance review and appraisal system (OPRAS), indicating that participation in ward rounds matter is mandatory and is part of the assessment if they participate. Inclusion of CPS in OPRAS has helped pharmacists to participate in the ward rounds and other clinical activities in the hospitals.” National Hospital Head of Pharmacy Unit.*
  - **Recognition of the provision of CPS by the Pharmacy Act and Regulations in Tanzania**
    Participants said that the current Pharmacy Act and regulations of Tanzania recognize CPS as one of the responsibilities of pharmacists, making it mandatory for pharmacists to be involved in the provision of primary healthcare directly to patients.
    *"Concerning the guidelines, I agree that our pharmacy law recognizes the presence of the clinical pharmacy profession. This necessitates pharmacists’ training for provision of CPS, especially at the level of bachelor’s degree in the universities. Therefore, laws and regulations are available to support the implementation of CPS in the hospitals.” National Hospital Pharmacist.*

## Discussion

The study aimed to assess the barriers and facilitators towards the integration of the pharmacist in the provision of CPS in Tanzania. The study explored the perspectives of medical doctors, nurses, and pharmacists. Five themes emerged from this study: three for barriers (individual-related, health facility-related, and health system-related) and two for facilitators (individual and health system efforts).

The current study found that limited skills for the provision of CPS, lack of confidence among pharmacists, and poor communication skills among pharmacists affect their involvement in provision of CPS. Similarly a study done in Brazil reported that the fear and frustration of pharmacists due to their lack of clinical and communication skills interfere with their participation in the provision of CPS [15]. In Kuwait it is reported that the pharmacists’ lack of proper clinical training and poor appreciation of pharmacy services by the physician were among the barriers to adequate provision of CPS [16]. In Nigeria, it was reported that medical doctors’ dominance and resistance to pharmacists’ intervention were among the reasons for pharmacists not providing CPS [17].

In this study, the factors influencing the effective implementation of CPS were superiority and inferiority behavior of HCPs, shortage of pharmacists in health facilities, lack of guidelines, SOPs and capacity building for in-job pharmacists on how to offer CPS. These factors fit well in the reported categories of factors that influence provision of CPS, namely attitudinal, political, administrative, and technical [18]. Furthermore, Jonathan and colleagues studied factors influencing the implementation of CPS in China and reported thematically related barriers and facilitators as reported in this study [13].

To address the barriers to the effective provision of CPS, studies recommend improvement of the training of pharmacists by exposing pharmacy students to patients and working with other HCPs during pre-service training. Several studies that have been conducted in developing countries have recommended introduction of a Doctor of Pharmacy (PharmD) degree, which exposes the trainees to healthcare life and builds their knowledge and skills for the provision of CPS [12, 16, 19, 20]. To increase assertiveness and confidence and to build inter-professional relationships among HCPs, the use of tools for communication is recommended [7]. The recommended tools include Strengths, Weaknesses, Opportunities, and Threats (SWOT) analysis which explains to doctors and nurses the aim of the intervention, and Specific, Measurable, Achievable, Realistic, and Timely (SMART) goals which breakdown the aim of the intervention to HCPs which later increases their awareness. Thirdly, the Interest, Desire, Action (AIDA) model, which shares the intervention results and helps build the trust of medical doctors and nurses towards the intervention can be used [7].

This study demonstrated a shortage of pharmacists, lack of in-job training for pharmacists, SOPs and guidelines for the provision of CPS, schedules for pharmacists to attend ward-rounds and limited physical space for pharmacists to offer pharmaceutical interventions while dispensing affect the provision of CPS. The findings are similar to what is reported in Ethiopia and United Arab Emirates (UAE) in which shortage of skilled pharmacists, high workload, shortage of staff, and lack of time and motivation were reported as impediments to the effective provision of health care services [14, 21]. Elsewhere, lack of clear career path and poor financial and leadership support are reported to be affecting the effective implementation of CPS.

The provision of CPS by pharmacists to has proven to have benefits, including improving the appropriateness of medication prescribing, implementing antimicrobial stewardship, and reducing morbidity and mortality among patients [2]. Awareness regarding CPS should be ensured from the level of administration to the level of HCPs. Pharmacists should be encouraged to participate in ward rounds and clinical meetings with other HCPs in the hospitals. The study done to assess the communication between medical doctors and pharmacists found that the acceptance rate of pharmacists’ interventions by doctors ranged between 39 and 70% when the assessment was done online, while the acceptance rate was much higher at 88.4% when the assessment was conducted through face-to-face interviews [22, 23]. These findings indicate that if proper information is provided about the roles of pharmacists in patient care, medical doctors are willing to work with pharmacists in the provision of CPS. Nevertheless, on-job training is needed to build their competencies in pharmacotherapy which is core for provision of CPS.

This study demonstrated that the acceptability of CPS by other HCPs, the positive perception of the pharmacists towards the provision of CPS, the readiness of the administration to offer support to pharmacists, and the recognition of CPS by the Pharmacy Act and regulations are factors motivating integration of pharmacists in the provision of CPS. In Brazil, a study on factors influencing the provision of CPS reported that responsible regulatory authorities and the government, through the Ministry of Health, encourage pharmacists to participate in the provision of CPS [24]. Other studies have reported attitudes and mindsets, adequate resources and space, support and mentorship, and pharmacists’ role definition are the determinants of CPS provision by the pharmacists [1, 20]. Furthermore, pharmacists with adequate confidence and assertiveness integrate well with other HCPs in providing primary healthcare services to patients.

### Trust and rigor of the study

The study follows four criteria to ensure trustworthiness: credibility, dependability, confirmability, and transferability. Triangulation approaches of mixing participants with different experiences using FGDs and IDIs approach in data collection and using researchers with different professions in data collection and analysis ensured the rigorousness of the study. Furthermore, the use of Swahili interview guides, a common language used by researchers and participants, and the availability of transcripts in Swahili made the analysis more robust. Results are presented using themes, sub-themes, and quotes to display the participants’ thoughts rather than the researchers’ interpretation. For the transferability of the findings, a detailed process of study design, setting, and the whole process of data collection and analysis has been provided. The findings from this study can be usefully in improving provision of CPS in developing countries with healthcare system equivalent to Tanzania.

## Conclusion

The facilitators and barriers to the integration of pharmacists in the provision of CPS lie at the individual, health facility, and health system levels. Acceptability of CPS by other HCPs and reliable administrative support are the key facilitators while limited skills, poor communication, shortage of staff, and lack of SoPs and guidelines are the main barriers to CPS provision. Therefore, the study recommends in-job pharmacists training, fostering teamwork among HCPs, developing CPS SoPs, and guidelines, and increasing the number of pharmacists per facility.

## Data Availability

The datasets used and/or analyzed during the current study are available from the corresponding author on reasonable request.

## Abbreviations

BMC: Bugando Medical Center
BMH: Benjamini Medical Hospital
CPS: Clinical Pharmacy Services
FGD: Focus Group Discussion
HCPs: Healthcare Providers
IDIs: In-Depth Interviews
IOM: Institute of Medicine
MNH: Muhimbili National Hospital
MUHAS: The Muhimbili University of Health and Allied Sciences
MZRH: Mbeya zonal referral hospital
REC: Research Ethical Committee
SoPs: Standard Operating Procedure
USA: United States of America
